# C-Reactive Protein to Prealbumin Ratio (CPR): A Novel Inflammatory-Nutritional Prognostic Factor for Predicting Cancer-Specific Survival (CSS) and Overall Survival (OS) in Patients with Resectable Esophageal Squamous Cell Carcinoma

**DOI:** 10.1155/2019/4359103

**Published:** 2019-07-14

**Authors:** Ji-Feng Feng, Liang Wang, You-Hua Jiang, Xun Yang

**Affiliations:** ^1^Department of Thoracic Oncological Surgery, Institute of Cancer Research and Basic Medical Sciences of Chinese Academy of Sciences, Cancer Hospital of University of Chinese Academy of Sciences, Zhejiang Cancer Hospital, No.38 Guangji Road, Hangzhou 310022, China; ^2^Key Laboratory Diagnosis and Treatment Technology on Thoracic Oncology, No. 38 Guangji Road, Zhejiang Province, Hangzhou 310022, China

## Abstract

**Background:**

The inflammation and nutrition play an important role in prognosis. A novel index combined with inflammatory and nutritional biomarkers, named C-reactive protein (CRP) to prealbumin (PALB) ratio (CPR), was initially reported to predict the prognosis in resectable esophageal squamous cell carcinoma (ESCC).

**Patients and Methods:**

A retrospective study was conducted including 346 resectable ESCC patients. The X-tile program was used to confirm the optimal cut-off value. The Kaplan-Meier methods and Cox regression analyses were performed to analyze the cancer-specific survival (CSS) and overall survival (OS).

**Results:**

The optimum cut-off point was 0.03 for CPR. Patients with a high level of CPR (> 0.03) were associated with poor CSS (12.0% vs. 43.0%,* P*<0.001) and OS (11.2% vs. 40.7%,* P*<0.001). Multivariate analyses revealed that CPR was an independent predictor in resectable ESCC patients (CSS,* P*=0.008; OS,* P*=0.007).

**Conclusion:**

This study, to the best of our knowledge, is the first to investigate prognostic role of CPR in patients with ESCC. Our retrospective observations indicate that CPR, with the optimal cut-off value of 0.03, is a useful potential predictor in resectable ESCC patients.

## 1. Introduction

Esophageal cancer (EC) is one of the prevalent cancers worldwide, whose incidences vary widely in different countries and regions, with approximately 53.8% and 51.9% of all ECs occurring and dying in China [1]. The major kind of pathological type (90%-95%) is esophageal squamous cell carcinoma (ESCC) in China [2, 3]. Radical esophagectomy remains the most effective therapy for patients with EC. However, the prognosis for EC remains poor [4, 5]. Therefore, it is very important to find more and more useful and effective prognostic indicators for patients with EC.

The inflammation and nutrition are associated with cancer prognosis [6]. As the most sensitive inflammatory biomarker, C-reactive protein (CRP) has been confirmed in a series of cancers to predict the prognosis, including EC [7–9]. Albumin (ALB) is an important nutritional biomarker. Several studies over the past few years have revealed that ALB serves as a prognostic factor for predicting prognosis in patients with EC [10, 11]. Over the recent years, a new index (combined with inflammatory and nutritional biomarkers) named CRP to ALB ratio (CAR) was reported to predict the prognosis for patients with EC [12, 13]. Additionally, recent studies reported that prealbumin (PALB), serving as another important biomarker for nutritional status, is more sensitive to malnutrition than ALB [14, 15].

It is commonly recognized that CRP and PALB are both cheap and simple serum biomarkers which could be conducted in daily clinical practices. However, to our knowledge, no study has assessed the prognostic role of CPR (CRP/PALB ratio) in ESCC patients so far. Thus, the aim of our study was initially to explore the prognostic role of CPR for predicting prognosis with the optimal cut-off value in resectable ESCC patients.

## 2. Patients and Methods

### 2.1. Patients

From January 2007 to December 2010 in the Department of Thoracic Oncological Surgery, a retrospective study involving 346 resectable ESCC patients was conducted, along with the confirmation of levels of serum CRP, ALB, and PALB one week before surgery. Patients who received preoperative treatment, such as chemotherapy and/or radiotherapy, those who suffered from any form of inflammatory diseases or infections (acute or chronic) or systemic diseases, and those diagnosed with distant metastases were excluded. Written informed consent for the collection of specimen and other medical information were obtained from all patients before surgery. The current study was approved by the Ethics Committees of Zhejiang Cancer Hospital (IRB Approval No. IRB-2018-130).

### 2.2. Treatment and Follow-Up

The standard esophagectomy includes the Ivor Lewis procedure (for patients with ESCC in the middle or lower third) and McKeown procedure (for patients with ESCC in the upper third) [16, 17]. The two-field thoracoabdominal lymphadenectomy was the major method of lymphadenectomy [18]. Follow-up was performed in regular intervals, and the follow-up results were obtained through reviews of the hospital records and outpatient visit.

### 2.3. Data Collection

The main clinical characteristics, such as age, gender, tumor length and location, vessel invasion, differentiation, TNM stage, and serum CRP, ALB, and PALB, were collected in our medical records. The TNM stage in this study was in accordance with the 7th AJCC/UICC TNM staging system [19]. The levels of CRP, ALB, and PALB were obtained within one week before surgery. CAR was defined as CRP to ALB ratio. CPR was defined as CRP to PALB ratio. The data used to support the findings of this study are included within the article.

### 2.4. Statistical Analysis

The X-tile program was performed to calculate the optimum cut-off points for CRP, ALB, PALB, CAR, and CPR [20]. The chi-squared test was utilized to analyze the clinical characters grouped by CPR. Besides, the Kaplan-Meier method and Cox regression analyses were utilized to analyze the cancer-specific survival (CSS) and overall survival (OS). The areas under the curve (AUC) for CPR, CAR, CRP, ALB, and PALB were calculated and compared by the receiver operating characteristic (ROC) curve. Statistical analyses were conducted with SPSS 20.0 (SPSS Inc., Chicago, IL, USA) and MedCalc 15.2 (MedCalc Software bvba, Ostend, Belgium).

## 3. Results

### 3.1. Patient Characteristics

**Figure 1:**
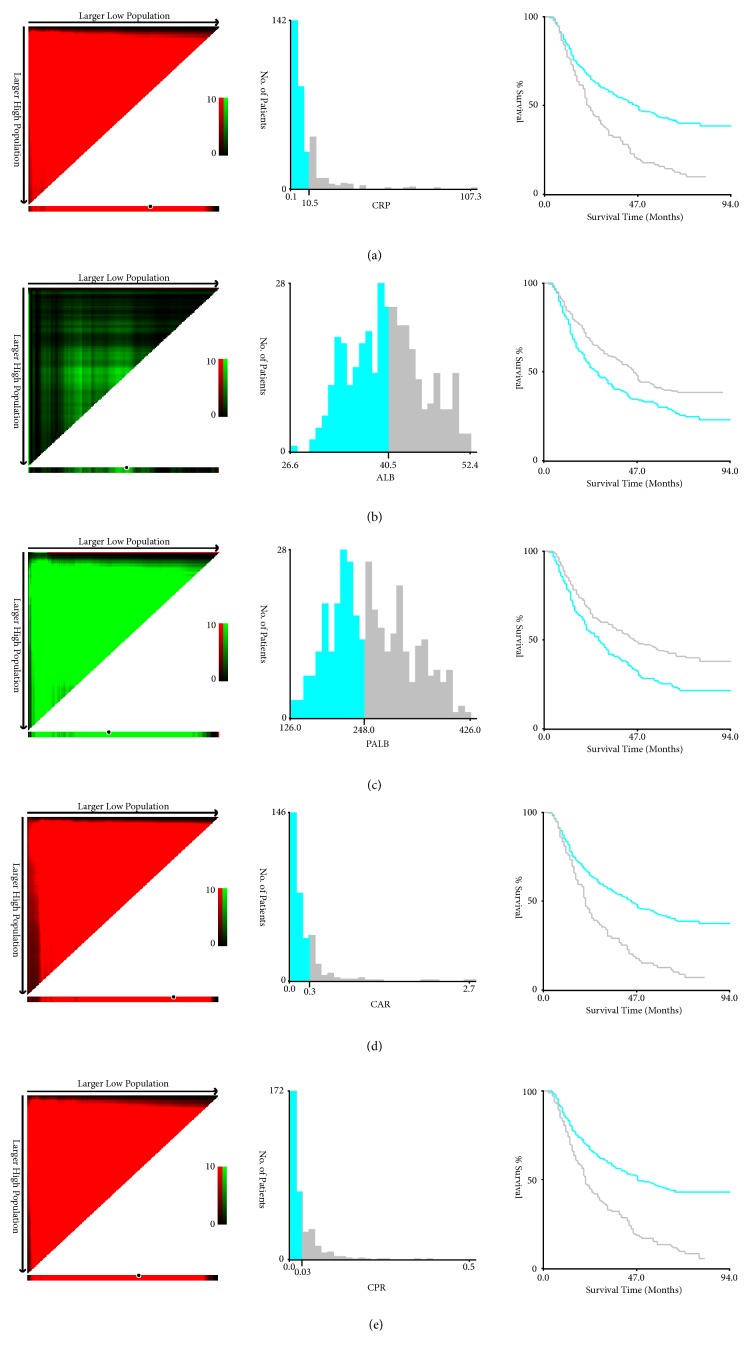
*X-tile analyses*. The optimum cut-off points according to the X-tile program for CRP (a), ALB (b), PALB (c), CAR (d), and CPR (e) were 10.5 mg/L, 40.5 g/L, 248 mg/L, 0.3, and 0.03, respectively.

There were 76 (22.0%) women and 270 (78.0%) men in all 346 patients. The mean values for CRP, ALB, PALB, CAR, and CPR were 8.55 ± 12.18 mg/L (range 0.10-107.34 mg/L), 40.3 ± 5.3 g/L (range 26.6-52.4 g/L), 260 ± 65 mg/L (range 126-426 mg/L), 0.22 ± 0.34 (range 0.002-2.670), and 0.0365 ± 0.0539 (range 0.0003-0.4993), respectively. The optimum cut-off points according to the X-tile program for CRP, ALB, PALB, CAR, and CPR were 10.5 mg/L, 40.5 g/L, 248 mg/L, 0.3, and 0.03, respectively (Figure 1).

Patients then were divided into two groups (high and low group) for further analyses (CPR ≤0.03 and CPR >0.03). The clinicopathologic characters regarding CPR and other clinical characters were shown in Table 1. Compared with the patients with CPR ≤ 0.03, patients with the CPR > 0.03 had even closer association with vessel invasion (*P*=0.025), TNM stage (*P*=0.035), and other indices (CRP,* P*<0.001; ALB,* P*=0.037; PALB,* P*<0.001; CAR,* P*<0.001).

**Figure 2:**
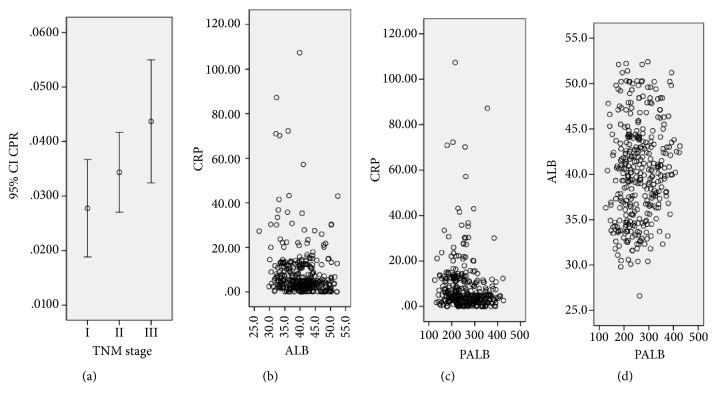
Significant differences were found between TNM I and TNM III (P=0.030) (a). Negative correlations between CRP and ALB (b) and CRP and PALB (c), respectively, and positive correlations between ALB and PALB (d) were found.

The mean values grouped by TNM stage for CPR were 0.0278 ± 0.0421, 0.0343 ± 0.0398, and 0.0437 ± 0.0680, respectively, with significant differences between TNM I and TNM III (*P*=0.030), but no significant differences regarding the CPR were found between the TNM I and TNM II (*P*=0.386) and TNM II and TNM III (*P*=0.166) (Figure 2(a)). Negative correlations between CRP and ALB (r* *=* *−0.166,* P *=* *0.002, Figure 2(b)) and CRP and PALB (r* *=* *−0.134,* P *=0* *.013, Figure 2(c)), respectively, and positive correlations between ALB and PALB (r* *=* *0.119,* P *=0.027, Figure 2(d)) were found in our study.

### 3.2. Cancer-Specific Survival and Overall Survival Analyses

**Figure 3:**
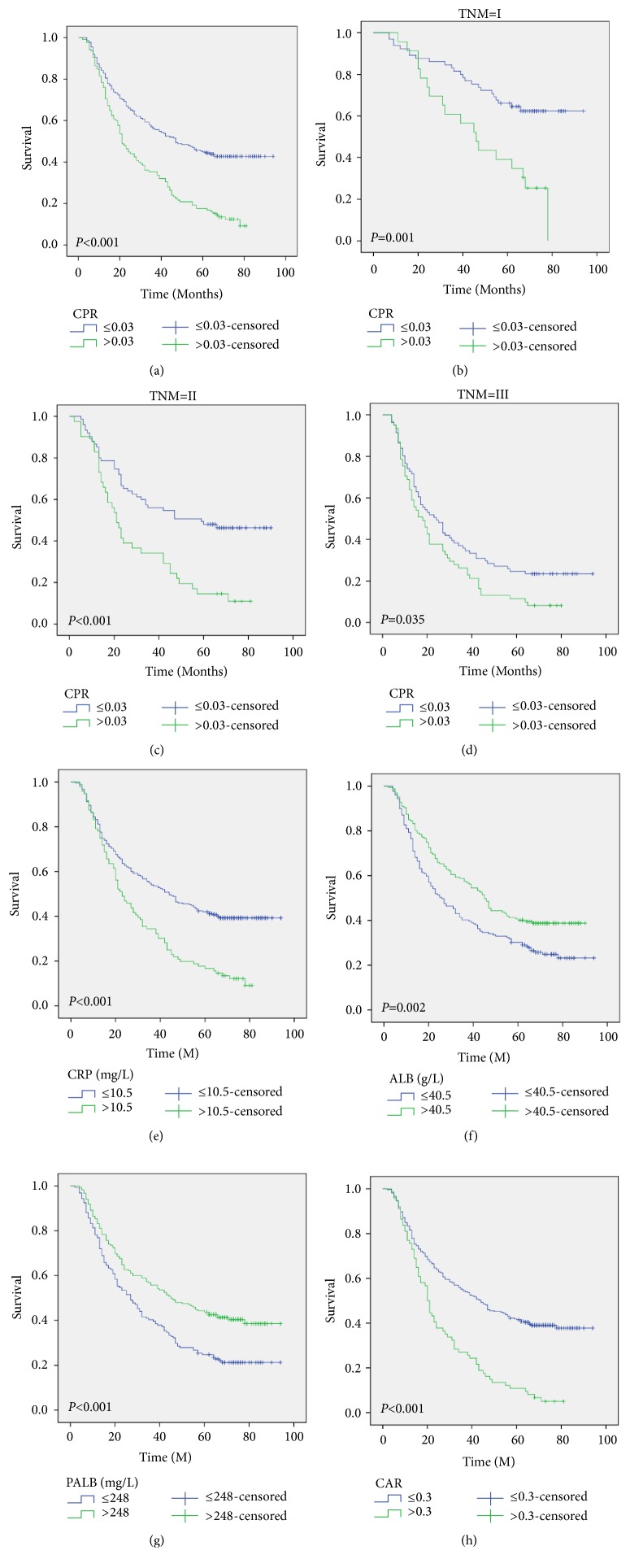
*CSS analyses*. Patients with a high level of CPR (>0.03) were associated with poor CSS (P<0.001) (a). CPR was also significantly associated with CSS based on TNM stage (b–d). There were also significant differences for patients grouped by CRP (e), ALB (f), PALB (g), and CAR (h).

**Figure 4:**
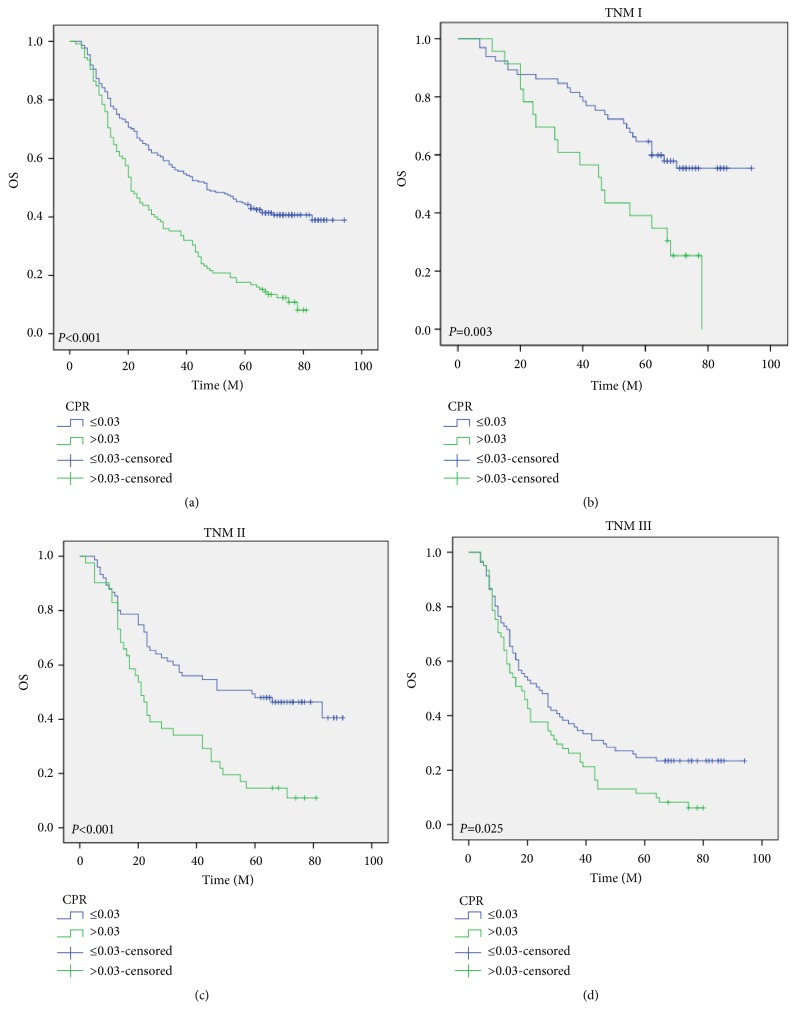
*OS analyses*. Patients with a high level of CPR (>0.03) were associated with poor OS (P<0.001) (a). CPR was also significantly associated with OS based on TNM stage (b–d).

Patients with a high level of CPR (>0.03) were associated with poor CSS (*P*<0.001). To be more specific, the 5-year CSS was 12.0% in patients with CPR >0.03 and 43.0% in those with CPR ≤0.03 (Figure 3(a)). It was revealed in subgroup analyses based on TNM stage that CPR was also significantly associated with CSS (Figures 3(b)–3(d)). The 5-year CSS were also significantly different for patients grouped by CRP (39.6% vs. 11.5%,* P*<0.001), ALB (25.1% vs. 38.9%,* P*=0.002), PALB (21.7% vs. 40.5%,* P*<0.001), and CAR (39.0% vs. 5.4%,* P*<0.001) (Figures 3(e)–3(h)). Patients with a high level of CPR (> 0.03) were associated with poor OS (11.2% vs. 40.7%,* P*<0.001) (Figure 4(a)). It was revealed that CPR was also significantly correlated with OS in the subgroup analyses based on TNM stage (Figures 4(b)–4(d)).

### 3.3. Cox Regression Analyses

It is generally recognized that several factors, such as tumor length, vessel invasion, CRP, ALB, PALB, CAR, CPR, and TNM stage, were significantly associated with CSS in univariate analyses (Table 2). However, we found that CPR (HR=1.630,* P*=0.008) was an independent predictor for CSS in multivariate analyses (Table 2). Multivariate analyses regarding OS in patients with ESCC were also performed. It was revealed that CPR was also an independent predictor (Table 3).

### 3.4. ROC Analyses

**Figure 5:**
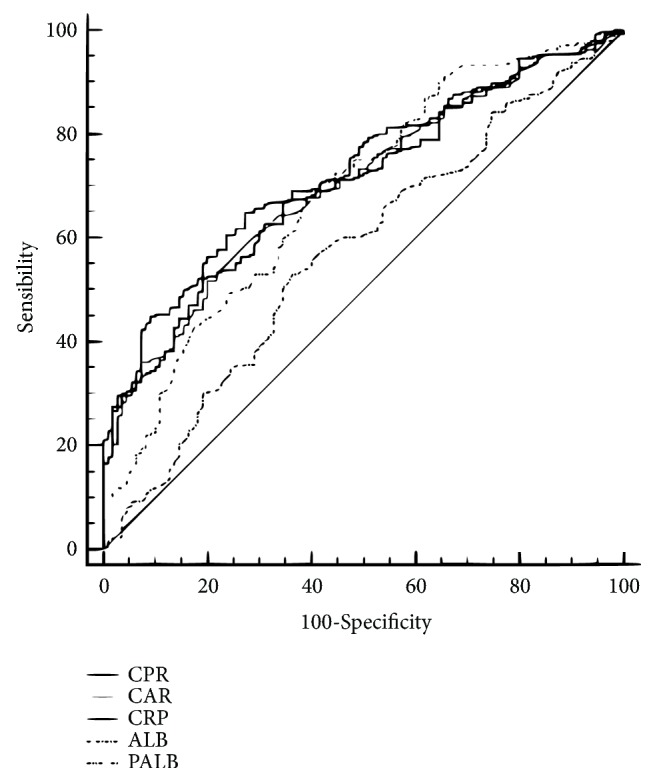
*ROC analyses*. The AUC area of the CPR (0.728) was higher than the areas of CAR (0.702), CRP (0.702), ALB (0.573), and PALB (0.686) for all the ESCC patients.

The AUC area of the CPR (0.728, 95% CI: 0.678-0.774) was higher than that of CAR (0.702, 95% CI: 0.651-0.750), CRP (0.702, 95% CI: 0.651-0.750), ALB (0.573, 95% CI: 0.519-0.625), and PALB (0.686, 95% CI: 0.635-0.735) for all the ESCC patients (Figure 5). Comparison of AUC areas for the prognostic factors in ESCC was shown in Table 4.

## 4. Discussion

To the best of our knowledge, this is the first study to investigate the prognostic role of CPR in patients with ESCC. Our study demonstrated some important findings: (1) CPR was a strong predictor of CSS and OS; (2) CPR, instead of CAR, CRP, ALB, or PALB, was a useful independent predictive indicator.

Related studies have shown that the presence of systematic inflammatory response and malnutrition are responsible for the poor prognosis in patients with cancers [6, 21]. CRP is the most sensitive inflammatory biomarker. However, the prognostic role of CRP remains controversial in EC [9, 22]. It was revealed in a meta-analysis conducted by us recently that CRP was significantly associated with overall survival in EC [23]. Recent published studies have reported that ALB was a nutritional factor, which reflected the nutritional status in several cancers, including EC [10, 11]. Furthermore, Xu* et al*. [12] and Wei* et al*. [13] reported that CAR is significantly associated with the prognosis in ESCC patients [12, 13]. Significant differences for patients grouped by CRP (39.6% vs. 11.5%,* P*<0.001), ALB (25.1% vs. 38.9%,* P*=0.002), and CAR (39.0% vs. 5.4%,* P*<0.001) were found in our study. However, CRP, ALB, and CAR were not independent predictive indicators.

Currently, as a serum index for the assessment on nutritional status, PALB has become a research focus, with it being reported as another important biomarker for nutritional status in recent studies, which is more sensitive to malnutrition than ALB [14, 15]. Recently, Li* et al*. [24] reported that CPR was independently correlated with hospital mortality. Lu* et al*. [25] revealed that the predictive value of CPR is significantly better than other biomarkers in the recurrence of gastric cancer. However, to our knowledge, no study has assessed the prognostic role of CPR in ESCC patients so far. Additionally, the predictive value between CPR (CRP/PALB) and CAR (CRP/ALB) remains unknown. Therefore, we conducted the current study to explore the prognostic value of CPR with the optimal cut-off value in resectable ESCC patients. In our results, patients with a high level of CPR (>0.03) were associated with poor CSS and OS (P<0.001). Even more, we found that CPR, instead of CAR, was an independent predictor for CSS and OS in multivariate analyses.

Currently, PALB, serving as an important biomarker for nutritional status, has become a research focus due to shorter half-life than ALB [26, 27]. Additionally, ALB is not a nutritional biomarker if malnutrition develops in a short time. Furthermore, the weight loss that was used to define malnutrition negatively correlated with PALB, but not with ALB [15]. Therefore, PALB is a more sensitive biomarker than ALB to assess the nutritional status in patients with ESCC. It is generally recognized that CRP and PALB are both routinely tested serum enzymes in daily clinical practices, which makes them easily available. Therefore, we have firstly explored the prognostic value of CPR in patients with ESCC in the current study. Besides, it is commonly argued by plenty of researchers that CRP and PALB may be influenced by a variety of other noncancer related conditions, and the potential basis could be decreased by the CRP to PALB ratio (CPR).

Another index for assessing nutritional status was BMI. Obesity is showing a rising trend worldwide with the improvement of living standards. It had been reported that the BMI was associated with the prognosis in EC [28]. This study suggested that BMI exerted a significant role in CSS and OS, with BMI being an independent factor for predicting CSS and OS in ESCC patients (*P*<0.001).

Limitations should be acknowledged in this study. The study is mainly limited by its retrospective character and the relatively small samples in a single center. Moreover, patients who received preoperative treatment, such as chemotherapy and/or radiotherapy, were excluded, which might influence the result of this study. Moreover, it is commonly acknowledged that neoadjuvant treatment will generate a side effect on CRP, ALB, and PALB. However, neoadjuvant treatment can improve cancer prognosis for locally advanced ESCC, but not for ESCC at the early stage [29, 30].

## 5. Conclusions

The study is the first time for us to identify (CRP/PALB) CPR and determine its prognostic value in ESCC patients undergoing esophagectomy. Our results revealed that CPR was an effective and independent predictor in resectable ESCC patients with the optimum cut-off point of 0.03.

## Figures and Tables

**Table 1:** Comparison of baseline clinical characteristics based on CPR in ESCC.

	Cases (n, %)	CPR		P-value
		≤ 0.03 (n, %)	> 0.03 (n, %)	
Age (years)				0.108
≤60	199 (57.5)	120 (54.3)	79 (63.2)	
>60	147 (42.5)	101 (45.7)	46 (36.8)	
Gender				0.077
Female	76 (22.0)	42 (19.0)	34 (27.2)	
Male	270 (78.0)	179 (81.0)	91 (72.8)	
Tumor length (cm)				0.242
≤ 3.0	96 (27.7)	66 (29.9)	30 (24.0)	
> 3.0	250 (72.3)	155 (70.1)	95 (76.0)	
Tumor location				0.179
Upper	18 (5.2)	11 (5.0)	7 (5.6)	
Middle	164 (47.4)	97 (43.9)	67 (53.6)	
Lower	164 (47.4)	113 (51.1)	51 (40.8)	
Vessel invasion				0.025
Negative	289 (83.5)	192 (86.9)	97 (77.6)	
Positive	57 (16.5)	29 (13.1)	28 (22.4)	
Differentiation				0.750
Well	47 (13.6)	32 (14.5)	15 (12.0)	
Moderate	230 (66.5)	144 (65.2)	86 (68.8)	
Poor	69 (19.9)	45 (20.3)	24 (19.2)	
TNM stage				0.035
I	88 (25.4)	65 (29.4)	23 (18.4)	
II	116 (33.5)	75 (33.9)	41 (32.8)	
III	142 (41.1)	81 (36.7)	61 (48.8)	
CRP (mg/L)				<0.001
≤ 10.5	250 (72.3)	219 (99.1)	31 (24.8)	
> 10.5	96 (27.7)	2 (0.9)	94 (75.2)	
ALB (g/L)				0.037
≤ 40.5	179 (51.7)	105 (47.5)	74 (59.2)	
> 40.5	167 (48.3)	116 (52.5)	51 (40.8)	
PALB (mg/L)				<0.001
≤ 248	161 (46.5)	81 (36.7)	80 (64.0)	
> 248	185 (53.5)	140 (63.3)	45 (36.0)	
CAR				<0.001
≤ 0.3	272 (78.6)	221 (100)	51 (40.8)	
> 0.3	74 (21.4)	0 (0)	74 (59.2)	

ESCC: esophageal squamous cell carcinoma; CRP: C-reactive protein; PALB: prealbumin; ALB: albumin; CPR: C-reactive protein to prealbumin ratio; CAR: C-reactive protein to albumin ratio; TNM: tumor, node, metastasis.

**Table 2:** Univariate and multivariate analyses of CSS in ESCC patients.

	Univariate analysis	P-value	Multivariate analysis	P-value
	HR (95% CI)		HR (95% CI)	
Age (years)		0.584		
≤ 60	1.000			
> 60	0.930 (0.717-1.206)			
Gender		0.530		
Female	1.000			
Male	1.105 (0.809-1.510)			
Tumor length (cm)		0.001		
≤ 3.0	1.000			
> 3.0	1.634 (1.208-2.211)			
Tumor location		0.845		
Upper	1.000			
Middle	1.160 (0.624-2.156)	0.638		
Lower	1.197 (0.644-2.222)	0.569		
Vessel invasion		0.003		
Negative	1.000			
Positive	1.636 (1.187-2.255)			
Differentiation		0.075		
Well	1.000			
Moderate	1.247 (0.834-1.864)	0.282		
Poor	1.660 (1.045-2.638)	0.032		
CRP (mg/L)		<0.001		
≤ 10.5	1.000			
> 10.5	1.896 (1.450-2.479)			
ALB (g/L)		0.002		
≤ 40.5	1.000			
> 40.5	0.669 (0.517-0.867)			
PALB (mg/L)		<0.001		
≤ 248	1.000			
> 248	0.613 (0.474-0.792)			
CPR		<0.001		0.008
≤ 0.03	1.000		1.000	
> 0.03	2.116 (1.634-2.739)		1.630 (1.135-2.342)	
CAR		<0.001		0.052
≤ 0.3			1.000	
> 0.3			1.485 (0.997-2.212)	
Adjuvant therapy	1.000	0.329		
No	1.149 (0.870-1.517)			
Yes				
TNM stage		<0.001		<0.001
I	1.000		1.000	
II	1.804 (1.237-2.631)	0.002	1.628 (1.111-2.387)	0.012
III	3.067 (2.150-4.373)	<0.001	2.559 (1.786-3.667)	<0.001
BMI (kg/m2)				
≥ 20	1.000	<0.001	1.000	<0.001
< 20	2.006 (1.551-2.595)		1.877 (1.444-2.440)	

ESCC: esophageal squamous cell carcinoma; CRP: C-reactive protein; PALB: prealbumin; ALB: albumin; CPR: C-reactive protein to prealbumin ratio; CAR: C-reactive protein to albumin ratio; TNM: tumor, node, metastasis; BMI: body mass index; CI: confidence interval; HR: hazard ratio.

**Table 3:** Multivariate analyses of OS in patients with ESCC.

	HR (95% CI)	P-value
TNM stage		
II vs. I	1.518 (1.048-2.199)	0.027
III vs. I	2.386 (1.685-3.379)	<0.001
BMI (kg/m2) (≤ 20 vs. > 20)	1.843 (1.422-2.388)	<0.001
CPR (> 0.03 vs. ≤ 0.03)	1.630 (1.140-2.322)	0.007
CAR (> 0.3 vs. ≤ 0.3)	1.474 (0.992-2.189)	0.055

ESCC: esophageal squamous cell carcinoma; OS: overall survival; TNM: tumor, node, metastasis; BMI: body mass index; CPR: C-reactive protein to prealbumin ratio; CAR: C-reactive protein to albumin ratio; CI: confidence interval; HR: hazard ratio.

**Table 4:** Comparison of AUC areas for the prognostic factors in ESCC.

	AUC	95% CI	P-value
CPR	0.728	0.678-0.774	Reference
CAR	0.702	0.651-0.750	0.0015
CRP	0.702	0.651-0.750	0.0008
ALB	0.573	0.519-0.625	0.0001
PALB	0.686	0.635-0.735	0.2161

ESCC: esophageal squamous cell carcinoma; CRP: C-reactive protein; PALB: prealbumin; ALB: albumin; CPR: C-reactive protein to prealbumin ratio; CAR: C-reactive protein to albumin ratio; AUC: area under the curve.

## Data Availability

The data used to support the findings of this study are included within the Supplementary Materials file.

## Supplementary Materials

Supplementary Materials The data used to support the findings of this study.
